# Biotic Interactions Are More Important than Propagule Pressure in Microbial Community Invasions

**DOI:** 10.1128/mBio.02089-20

**Published:** 2020-10-27

**Authors:** Michaeline B. N. Albright, Sanna Sevanto, La Verne Gallegos-Graves, John Dunbar

**Affiliations:** a Bioscience Division, Los Alamos National Laboratory, Los Alamos, New Mexico; b Earth and Environmental Sciences Division, Los Alamos National Laboratory, Los Alamos, New Mexico; Corporación CorpoGen

**Keywords:** microbiome engineering, probiotics, ecosystem manipulation, invasion biology, bacterial traits, ecosystem functioning, fungal traits, microbial composition

## Abstract

Microbial probiotics are intended to improve functions in diverse ecosystems, yet probiotics often fail to establish in a preexisting microbiome. This is a species invasion problem. The relative importance of the two major factors controlling establishment in this context—propagule pressure (inoculation dose and frequency) and biotic interactions (composition of introduced and resident communities)—is unknown. We tested the effect of these factors in driving microbial composition and functioning following 12 microbial community invasions (e.g., introductions of many microbial invaders) in microcosms. Ecosystem functioning over a 30-day postinvasion period was assessed by measuring activity (respiration) and environment modification (dissolved organic carbon abundance). To test the dependence on environmental context, experiments were performed in two resource environments. In both environments, biotic interactions were more important than propagule pressure in driving microbial composition and community function, but the magnitude of effect varied by environment. Successful invaders comprised approximately 8% of the total number of operational taxonomic units (OTUs). Bacteria were better invaders than fungi, with average relative abundances of 7.4% ± 6.8% and 1.5% ± 1.4% of OTUs, respectively. Common bacterial invaders were associated with stress response traits. The most resilient bacterial and fungal families, in other words, those least impacted by invasions, were linked to antimicrobial resistance or production traits. Illuminating the principles that determine community composition and functioning following microbial invasions is key to efficient community engineering.

## Abstract (precis)

With increasing frequency, humans are introducing new microbes into preexisting microbiomes to alter functioning. Example applications include modification of microflora in human guts for better health and those of soil for food security and/or climate management. Probiotic applications are often approached as trial-and-error endeavors and have mixed outcomes. We propose that increased success in microbiome engineering may be achieved with a better understanding of microbial invasions. We conducted a microbial community invasion experiment to test the relative importance of propagule pressure and biotic interactions in driving microbial community composition and ecosystem functioning in microcosms. We found that biotic interactions were more important than propagule pressure in determining the impact of microbial invasions. Furthermore, the principles for community engineering vary among organismal groups (bacteria versus fungi).

## INTRODUCTION

The use of probiotics—microbial inoculants intended to manipulate microbial communities—to achieve desired functional outcomes is rapidly increasing (1). Examples include modifications of microflora in human guts for better human health, bioreactors for fuel production, and soil for improved plant performance and/or climate management (1–9). The effectiveness of this approach depends on the predictable establishment and persistence of introduced microbes, yet the parameters for successful introductions are unknown. Introduced microbes and their beneficial effects often fail to persist (1, 10–12). Consequently, microbial community manipulation continues to be a trial-and-error endeavor with low success rates, pointing to the need to understand the fundamental principles for successful microbiome engineering.

Microbiome engineering is related to invasion biology, which generally aims to understand factors controlling the success of invasive plant and animal species. In invasion biology, invasion success is determined by three main processes: (i) environmental filtering, (ii) propagule pressure, and (iii) biotic interactions (13, 14). Environmental filtering involves the general compatibility of an invader with a new environment (e.g., suitable temperature range). Propagule pressure encompasses dispersal potential, which determines which species are likely to spread to novel areas, either through natural or human-mediated movement. Components of propagule pressure, dose and frequency, describe the magnitude and pattern of arrival of invasive individuals (15). Propagule pressure is one of the most common explanatory factors of invasion success in microbial (10, 15–22) as well as macrobial invasion studies (14, 23). Biotic interactions encompass a range of interactions (e.g., competition, facilitation, or predation) that may occur between the invader and residents (24–26) and depend implicitly on the identity and number of taxa (i.e., composition). Often in invasion studies, broad characteristics such as composition, richness, and diversity are compared across resident communities and used to infer the importance of biotic interactions as a driving factor in invasion success (1, 11, 27–30). Richness and diversity reflect a likelihood of interactions because as the number of species increases, more interactions are possible.

The relative importance of the three processes remains unclear. Furthermore, success is often measured as the establishment of an invasive species, while the impacts of invaders on the composition and functioning of the larger community are not assessed (31, 32). Assessing the relative importance of these processes is hampered by the conventional focus on studies of one invader at a time. Although focusing on a single invader allows more control and more detailed investigation of underlying biology, the approach creates a risk of sampling bias, wherein researchers focus on organisms of interest or convenience, and the limited scope of diversity leads to eccentric conclusions. An alternative approach is to examine invasion events that involve a diverse natural assemblage of invaders. The natural mixing of entire microbial communities at ecosystem transition zones (33–35), coined microbial community coalescence, has been explored in a number of arenas (5), and yet focused studies of microbial invasion are rare (20, 30). A promising new approach to quantify factors leading to successful invasions is to introduce whole communities rather than individual taxa (36–39). Introducing complex communities tests many species invasions simultaneously and recapitulates natural microbial dispersal events such as rain or wind dispersal of soil microbes to plant litter on the soil surface (40). As multispecies manipulations are often unfeasible in macroecology due to the large scale, microbial research can richly inform invasion biology, just as it has impacted evolutionary biology (41).

**FIG 1:**
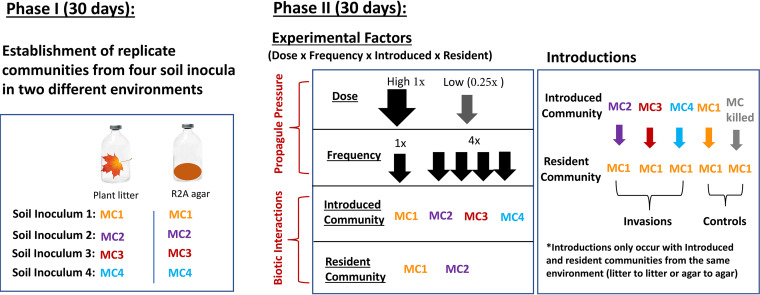
Experimental setup used to test factors driving composition and functional outcomes of microbial community invasions. In phase I, four microbial suspensions created from soils were used to inoculate microcosms in order to establish many replicates of complex communities in plant litter and R2A agar substrates. In phase II we conducted microbial community invasions, while varying four factors, including dose, frequency, and introduced and resident communities (see Materials and Methods for details).

We conducted a microbial community invasion experiment with 12 invasions to test the relative importance of propagule pressure (delivery parameters) and biotic interactions on microbial community composition and ecosystem functioning. We did not focus on specific types of biotic interactions. Rather, we use the term broadly and we use microbial community composition—the source of biotic interactions—as a metric of changes in the suite of interactions with invader and resident communities. We manipulated four factors: (i) inoculation dose, (ii) inoculation frequency, (iii) invader community composition, and (iv) resident community composition in a microcosm experiment. Since our goal was to decipher common rules for disparate microbiome engineering applications, manipulations were performed in microcosms with two distinct resource environments. One environment was R2A agar and the other was plant (Pinus ponderosa) litter on sand. R2A agar contains more labile carbon (C) and allows for homogenous mixing, while the litter environment contains more recalcitrant C and greater structural complexity. We preadapted invaders to each environment, minimizing environmental filtering as a test factor. During phase I of the experiment, we established four model microbial communities by inoculating microcosms with soil suspensions (Fig. 1). In phase II, we created 12 invasions by mixing phase I communities at different doses and frequencies (Fig. 1). During phase II, we measured carbon dioxide (CO_2_) production and dissolved organic carbon (DOC) abundance as metrics of ecosystem functioning (i.e., microbial activity and modification of the environment, respectively). Because propagule pressure is widely considered a primary determinant of invasion success (14, 23), we hypothesized that propagule pressure would play a larger role than biotic interactions in shaping microbial composition and ecosystem functioning.

## RESULTS

### Impacts of propagule pressure and biotic interactions on ecosystem functioning.

To assess principles across ecosystems, we examined the relative roles of propagule pressure and biotic interactions on ecosystem functioning in two resource environments: R2A agar and plant litter. Four invader communities (MC1, MC2, MC3, and MC4), two resident communities (MC1 and MC2), two doses (high and low), and two frequencies (high and low) were tested, with three replicate microcosms per treatment type (*n* = 240 total microcosms) (Fig. 1). We quantified the relative contribution of propagule pressure (i.e., dependence on dose and/or frequency) versus that of biotic interactions (i.e., dependence on initial community compositions of the residents and/or invaders) in driving variation in community composition and ecosystem functioning by using a multifactorial permutational multivariate analysis of variance (PERMANOVA)/ANOVA design followed by partitioning of variance (see Materials and Methods). Complete results from statistical tests are provided in Table S1 in the supplemental material.

10.1128/mBio.02089-20.2 TABLE S1 ANOVA and PERMANOVA results from complete and reduced models for factors driving variation in ecosystem functioning (CO_2_ and DOC accumulation) and bacterial and fungal richness and composition in litter and agar environments. Download Table S1, DOCX file, 0.6 MB. Copyright © 2020 Albright et al. 2020 Albright et al. This content is distributed under the terms of the Creative Commons Attribution 4.0 International license.

**FIG 2:**
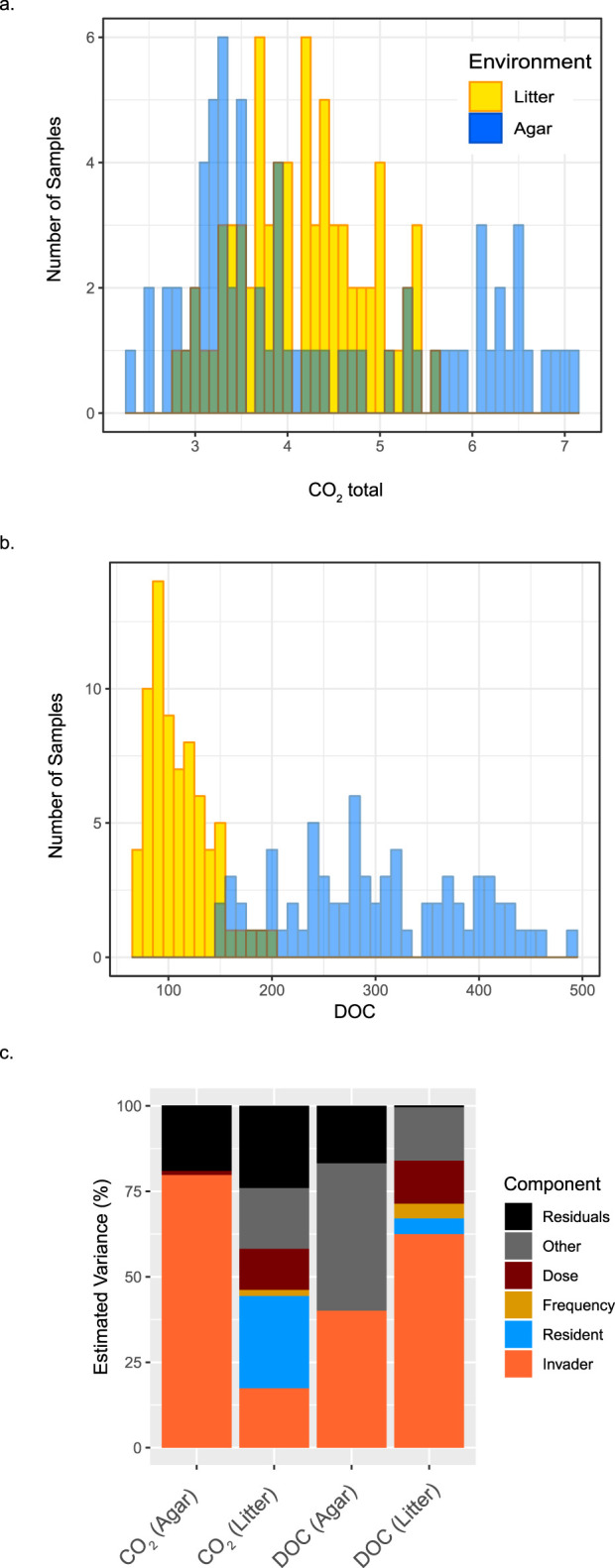
Histogram of the distribution of ecosystem functioning metrics, CO_2_ production (a) and DOC abundance (b), across phase II invaded microcosms (*n* = 144). Litter environment measures are shown in gold, and medium environment measures are shown in blue. (c) Impact of propagule pressure (dose and frequency) and biotic interactions (resident and introduced composition) in driving ecosystem functioning measured as total CO_2_ production and DOC abundance. Estimated variance was computed on reduced ANOVA models. Only significant main factors are shown, and the “other” component is a sum of significant interaction terms. Complete statistics are in Table S1 in the supplemental material.

Biotic interactions were the primary driver of variation in functioning, accounting for 7-fold (average) more variation than propagule pressure (Fig. 2c). Overall, total CO_2_ production across all invaded microcosms varied by 3.1-fold in the agar and 2.0-fold in the litter (Fig. 2a), while DOC abundance varied 3.3-fold and 3.1-fold (Fig. 2b). Propagule pressure did not impact functional outcomes in the agar environment, with the exception of the minor role of dose (1.2%) in driving variation in CO_2_ production. However, in litter, dose played a role in driving 12% of variance in both CO_2_ production and DOC (Fig. 2; Table S1), and the abundance of CO_2_ and DOC was greater in high- than low-dose samples (see Fig. S1a to d). Frequency accounted for minor variation in CO_2_ production (1.8%) and DOC abundance (4.3%) in the litter; DOC was higher in communities with four introductions than those with one introduction.

10.1128/mBio.02089-20.4 FIG S1 CO_2_ production across dose treatments in agar (a) and litter (b) environments. DOC accumulation across dose treatments in the agar (c) and litter (d) environments. CO_2_ production across invaded and control communities in the agar (e) and litter (f) environments. DOC accumulation across invaded and control communities in the agar (g) and litter (h) environments. Download FIG S1, EPS file, 1.7 MB. Copyright © 2020 Albright et al. 2020 Albright et al. This content is distributed under the terms of the Creative Commons Attribution 4.0 International license.

For biotic interactions, the impact of invader versus resident community composition on ecosystem functioning varied by environment (Fig. 2c). In the agar, the invader community composition accounted for 80% of variation in CO_2_ production and 40% of variation in DOC abundance, while the resident community composition did not contribute to variance in ecosystem functioning. In contrast, the contribution of the invaders and resident communities to CO_2_ variation in the litter were more even, accounting for 17% and 27% of variation, respectively. The invader community accounted for 62% of DOC variation in the litter, while the resident community played a much smaller role (5% of variation). In the agar, some invaded communities produced significantly more CO_2_ than controls, whereas in the litter, the average quantity of CO_2_ was not significantly different between invaded and control communities (Fig. S1e to f). Overall, invader MC3 had the greatest impact on ecosystem functioning (Fig. S1e to h). These results show that the potential to alter functioning by invasion depends on both the environment and the invader community composition.

### Impacts of propagule pressure and biotic interactions on community composition.

**FIG 3:**
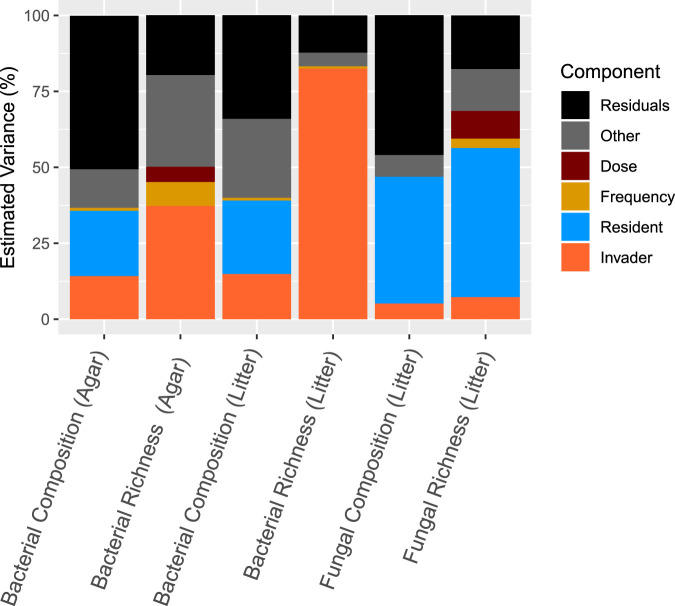
Impact of propagule pressure (dose and frequency) and biotic factors (resident and invader composition) in driving bacterial and fungal composition and richness in litter and agar environments. Estimated variance was computed on reduced ANOVA and PERMANOVA models. Only significant main factors are shown, and the “other” component is a sum of significant interaction terms. Complete statistics are in Table S1.

As with ecosystem functioning, in both environments, biotic interactions (invader and resident community) played a larger role, accounting for an average of 40-fold more variation than propagule pressure (dose and frequency) in shaping final community composition and richness (Fig. 3; Table S1). The minor role of propagule pressure was relatively greater in shaping richness than composition (Fig. 3). For richness, the influence of invasion frequency and dose varied depending on organism type (bacteria versus fungi) and environmental type. Bacterial richness was affected by invasion frequency, dose, and frequency-by-dose interactions in the agar environment (Fig. 3). Higher invasion dose and frequency drove higher bacterial richness in agar but not in litter (see Fig. S2a, b, d, and e). For fungi, higher dose invasions increased richness, whereas higher frequency invasions decreased richness (Fig. S2c and f).

10.1128/mBio.02089-20.5 FIG S2 Final richness across frequency treatments for bacteria in agar (a), bacteria in litter (b), and fungi in litter (c). Final richness across dose treatments for bacteria in agar (d), bacteria in litter (e), and fungi in litter (f). Download FIG S2, EPS file, 1.2 MB. Copyright © 2020 Albright et al. 2020 Albright et al. This content is distributed under the terms of the Creative Commons Attribution 4.0 International license.

The relative contribution of initial invader and resident community compositions to biotic interactions shaping final community composition was parsed by organism domain (bacteria versus fungi). For bacteria, approximately 15% of estimated variation in composition (i.e., beta-diversity) in both environments was driven by invaders, while residents drove approximately 23% of variation (Fig. 3; Table S1). In contrast, fungal residents played a larger role than invaders in driving community composition in litter, contributing to 42% (versus 5%) of estimated variance in composition (Fig. 3; Table S1). In the agar, levels of fungal communities collapsed to below sequence detection limits. Both bacterial and fungal community compositions were also impacted by a resident-by-invader community interaction (Table S1). Invaded bacterial community composition was significantly different from that of controls that accounted for necromass nutrient addition (e.g., MC1 plus killed-MC1) and the physical perturbation of the invasion procedure (e.g., resident-resident mix and MC1-MC1) (pairwise permutation multivariate ANOVAs). We observed similar trends for richness, with a greater impact of the invaders on bacterial richness and a greater impact of residents on fungal richness (Fig. 2; see also Fig. S5). Generally, invaded communities had higher bacterial richness and diversity than controls (i.e., resident plus killed or resident plus resident) (Fig. S3a, b, d, and e). Fungal richness and diversity were unaffected by invasion (Fig. S3c and f).

10.1128/mBio.02089-20.6 FIG S3 Richness of final invaded and control communities for bacteria in agar (a), bacteria in litter (b), and fungi in litter (c). Shannon diversity of final invaded and control communities for bacteria in agar (d), bacteria in litter (e), and fungi in litter (f). Download FIG S3, EPS file, 1.6 MB. Copyright © 2020 Albright et al. 2020 Albright et al. This content is distributed under the terms of the Creative Commons Attribution 4.0 International license.

As expected, the environment was a strong driver of composition. Bacterial communities differed by environment type (Fig. S4a), but after 60 days, bacterial richness was similar across the two environments (Fig. S4b). The levels of fungal communities collapsed in the agar; thus, analyses for fungal communities were performed only in the litter environment.

10.1128/mBio.02089-20.7 FIG S4 Bacterial community composition (a) and bacterial richness (b) across final samples colored by environment: litter (yellow) and agar (blue). Download FIG S4, EPS file, 0.7 MB. Copyright © 2020 Albright et al. 2020 Albright et al. This content is distributed under the terms of the Creative Commons Attribution 4.0 International license.

10.1128/mBio.02089-20.8 FIG S5 (a) Relative abundance of bacterial families across initial inoculum communities. (b) Nonmetric multidimensional scaling (NMDS) ordinations showing variability in bacterial community composition (Bray-Curtis dissimilarity) across initial resident communities. (c) Relative abundance of fungal orders across initial inoculum communities. (d) NMDS ordinations showing variability in fungal community composition (Bray-Curtis dissimilarity) across initial resident communities. Download FIG S5, JPG file, 0.3 MB. Copyright © 2020 Albright et al. 2020 Albright et al. This content is distributed under the terms of the Creative Commons Attribution 4.0 International license.

### Quantifying invasion success and identifying invader taxa.

**FIG 4:**
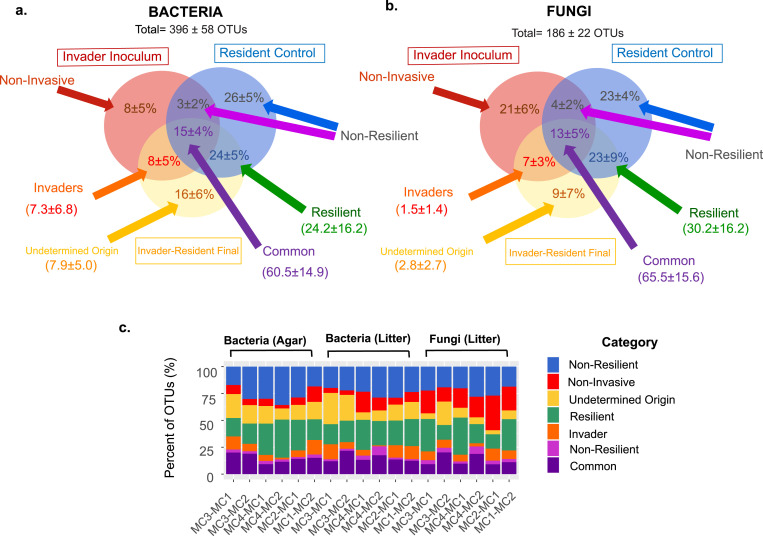
Percentage of OTUs that were invaders, resilient, common, noninvasive, or nonresilient across the 12 invasion events. Average OTU distributions for bacteria (a) and fungi (b) across phase I and phase II samples are shown in the Venn diagram circles. For the OTUs in each category, the total relative abundance of OTUs in the phase II invader-resident final communities is shown in parentheses. (c) Distribution of OTUs across categories for each individual invasion event. (Data shown in Table S2.)

To quantify invasion success and the characteristics of effective competitors, we categorized taxa as (i) invaders, (ii) noninvaders, (iii) resilient, (iv) nonresilient, (v) common, or (vi) undetermined, as defined in Materials and Methods. To do this, we assessed the presence of operational taxonomic units (OTUs) in the final invaded communities relative to those in the invader inoculum and resident community. To be more comprehensive, the resident community was represented as a composite of sequence data from the phase II initial resident, the final resident-resident, and the final killed-resident community samples (Fig. 4; see also Table S2).

10.1128/mBio.02089-20.3 TABLE S2 Summary statistics. Percent OTUs across phase I and phase II samples and the total relative abundance of OTUs in phase II invader-resident final communities. Data for each of the 12 unique invasion events (MC1-MC2, MC4-MC2, etc.) are an average from 100 rarefactions. Average, standard deviation, minimum, and maximum values across the 12 invasion events were calculated. In addition, data are shown for rarefaction cutoffs of 1,020/1,262 (bacteria/fungi), 5,000, and 10,000 sequences. Download Table S2, XLSX file, 0.1 MB. Copyright © 2020 Albright et al. 2020 Albright et al. This content is distributed under the terms of the Creative Commons Attribution 4.0 International license.

On average bacterial communities contained 396 ± 58 OTUs (Table S2). Across the 12 invasion events, the distributions of OTUs across categories were highly consistent. Bacterial invaders comprised 8% ± 5% of the total number of OTUs, with an average relative abundance of 7.4% ± 6.8% in invader-resident final samples. This is a conservative estimate, because 16% ± 6% of bacterial OTUs were of undetermined origin (i.e., OTUs found in the final invaded communities but undetected in either the resident or invader inoculum communities) (Fig. 4a and b). The undetermined OTUs were likely undetected in the invader inoculum, since the invader inoculum with 7,140 to 14,280 sequences (rarefied sequences × number of samples) had a weaker detection limit than the resident control with 17,340 to 27,540 sequences. More samples were sequenced for the resident control to limit false-positive classifications of taxa as “invaders” (Table S2). Resilient bacteria comprised 24% ± 5% of bacterial OTUs (Fig. 4). Common bacterial OTUs were 15% ± 4% (Fig. 4; Table S2). On average, fungal litter communities comprised 186 ± 22 OTUs. The distribution of OTUs across categories was very similar to that for bacteria, with the exception of an increase in noninvasive fungi, which was 21% ± 6% of fungal OTUs versus 8% ± 5% of bacterial OTUs (Fig. 4). In addition, while fungal invaders comprised 7% ± 3% of the OTUs, the average relative abundance was only 1.5% ± 1.4% in invader-resident final samples.

**FIG 5:**
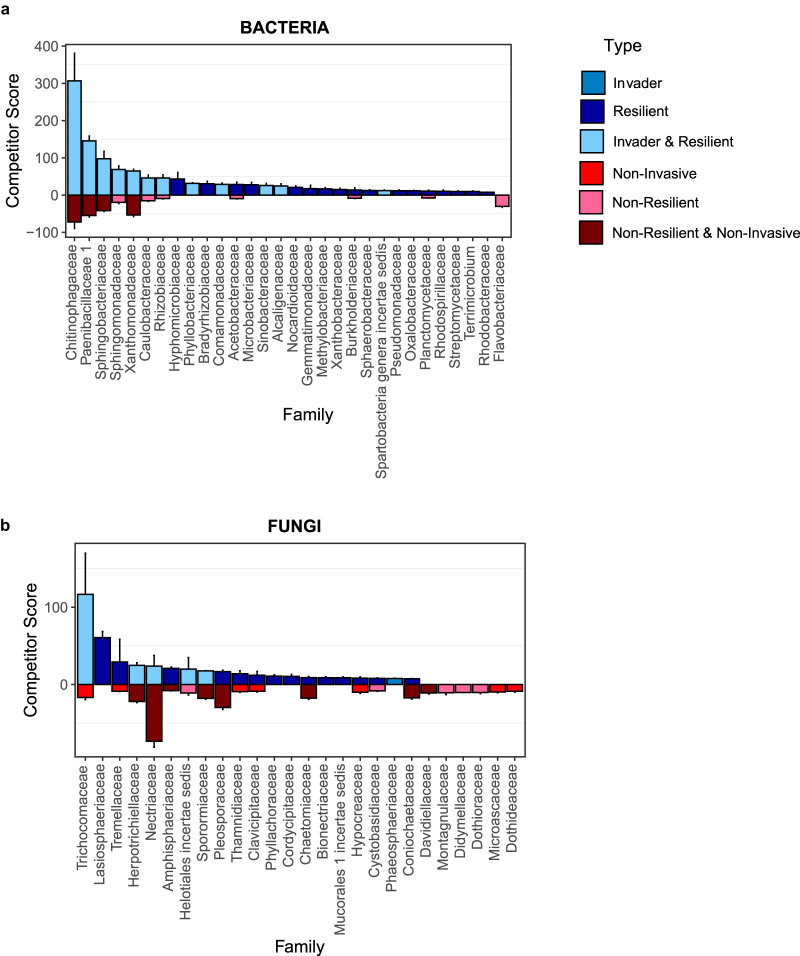
Competitor scores for bacterial (a) and fungal (b) families, shown as the square root of the calculated competitor score (see Materials and Methods). Strong competitors are shown in blue (invader, resilient, and invader & resilient), and weak competitors are shown in red (noninvasive, nonresilient, and nonresilient & noninvasive).

The competitive success of taxa was evaluated at the family level, based on the fraction of member OTUs that succeeded as invaders and/or as resilient residents (Fig. 5a). The strongest competitor bacterial families in both the agar and litter environments were both invasive and resilient and included *Chitinophagaceae*, *Paenibacillaceae*, *Sphingobacteriaceae*, and *Sphingomonadaceae*. Seventeen families were resilient across both environments. We identified 11 weak competitor families, including *Flavobacteriaceae*, which was nonresilient. Ten families were mixed, having both strong and weak competitors. For fungi, the most successful competitors were largely resilient, rather than invaders, and included *Lasiosphaeriaceae*, *Tremellaceae*, and *Amphisphaeriaceae* (Fig. 5b). Six families were exclusively categorized as weak competitors: *Davidiellaceae*, *Montagnulaceae*, *Didymellaceae*, *Dothioraceae*, *Microascaceae*, and *Dothideaceae*. An additional 14 families were categorized as mixed.

## DISCUSSION

Understanding of the principles for successful microbiome engineering across systems is needed to increase the effectiveness of probiotics. To our knowledge, this is the first study to test the relative importance of propagule pressure and biotic interactions in driving the variation in microbial community compositions and ecosystem functioning following a complex microbial community invasion event. In both macro- and microorganism studies of (mostly) single-organism invasions, increasing propagule pressure leads to increased establishment of invaders in both field and laboratory experiments (10, 15, 20, 21, 42–44). However, impacts of invaders on community composition and functioning have been largely neglected. In our study, propagule pressure was less important than biotic interactions in driving variation in microbial composition and ecosystem functioning (Fig. 2 and 3). Invasions with large numbers of diverse taxa compared to those by single taxa may increase the importance of biotic interactions relative to propagule pressure. In this scenario, resident communities must resist larger numbers of invaders, and invaders must compete not only with other invader taxa but also with residents. A few studies have found that groups of microbes have the potential to be both more robust and more productive than monocultures as inoculants (45–47). For example, fecal microbiome transplants (complex community) have been found to be more effective than a probiotic mix (simple community) for microbiome recovery after antibiotic disturbance (48). With a diverse invader community, the probability of including a taxon that has the ability to establish regardless of propagule pressure increases. This is supported by the observation that successful invaders in our study occurred in both high and low abundance in the invader inoculum, demonstrating a lack of dependence on propagule pressure. Our results suggest that research to improve probiotic manipulation of microbial communities would be better spent focusing on characteristics of inoculants and residents rather than on delivery dose and frequency.

Given the increasing evidence of strong links between microbial community composition and functioning (49–51), it is perhaps unsurprising that both were most strongly impacted by the same factor—biotic interactions. However, the degree of impact on functioning depended on the environment context (Fig. 5c). In the agar environment, the larger role of invader communities in driving changes in ecosystem functioning may be due to the loss of fungi, leading to a more prominent role of bacterial invaders. In contrast, litter fungi appeared to be the most important players in driving CO_2_ dynamics (see text and Fig. S6 in the supplemental material), and the resident litter communities played the greatest role in determining both final fungal composition and CO_2_ production (Fig. 2; Fig. 5c). Overall, our results confirm the potential to alter ecosystem functioning by community invasions, but the environment matters. Furthermore, understanding of the cause of the differences in invader impacts on ecosystem functioning between environments is an important focus for further research.

10.1128/mBio.02089-20.9 FIG S6 Correlations between bacterial richness and CO_2_ production (a), fungal richness and CO_2_ production (b), bacterial richness and DOC accumulation (c), and fungal richness and DOC accumulation (d). Colors represent environments with litter in yellow and agar in blue. Pearson’s correlations for each environment are shown. Nonmetric multidimensional scaling (NMDS) ordinations showing variability in 16S bacterial community composition (16S) of final communities (*n* = 212) (e) and fungal community composition (LSU) of final communities (*n* = 96) (f) using the Bray-Curtis dissimilarity metric. Points are colored by the most significant correlation with functioning, for bacteria, this is by high, mid, and low DOC in each environment type, while for fungi, by high, mid, and low CO_2_. Download FIG S6, EPS file, 2.4 MB. Copyright © 2020 Albright et al. 2020 Albright et al. This content is distributed under the terms of the Creative Commons Attribution 4.0 International license.

While biotic interactions were the strongest driver, the relative impact of initial invader compared to that of the resident community composition varied by organism type (Fig. 3). Previous research has largely focused on the role of increased resident community richness and diversity in reducing invasion success (1, 11, 27–30). Such studies attempt to identify the aspects of resident communities that are driving outcomes in contrast to the relative roles of residents and invaders in shaping final microbial richness and composition. This focus on resident communities is likely influenced by current engineering practices that routinely use simple invasions with only one or a few microbial “invaders” (11, 52–54). With complex community invasions, invaders were important in driving bacterial communities, whereas residents played a larger role in shaping fungal communities (Fig. 3). In addition, bacteria were better invaders than fungi (Fig. 3 and 4). Other studies looking at the effects of abiotic (environmental) perturbations on microbial communities have found that bacterial communities are generally less resistant to change than fungal communities (51, 55). More generally, bacterial and fungal community assembly has been found to differ (56–58). Factors such as growth rate, growth habit (unicellular versus filamentous), and resource utilization breadth have also been hypothesized to play a role in differences in bacterial versus fungal establishment (51, 59). We expect these factors are likely important in our system as well. Our results suggest that engineering fungal communities may be more difficult than engineering bacterial communities.

Understanding the characteristics that enable microbial invaders to establish in preexisting communities is a component of success in all probiotic engineering applications, but little is known about conducive traits (60). Characteristics such as dispersal ability, reproductive strategy, and growth form have been linked to invader success in macroorganisms (61–63). Most previous microbial invasion studies have risked bias by choosing invaders-of-interest *a priori* (20). An advantage of studying invasion principles with complex microbial communities is that it omits *a priori* selection and allows identification of a suite of successful invaders in a single experiment, facilitating the search for common invader characteristics. We identified a number of successful invader microbial families, and here we highlight several with known traits that may contribute to this success. In particular, *Sphingobacteriaceae* (*Bacteriodetes*) and *Sphingomonadaceae* (*Alphaproteobacteria*) stood out as bacterial invaders (Fig. 5a). Both these families are environmental bacteria capable of producing sphingolipids, a relatively uncommon microbial trait (64). Sphingolipids have been shown to play an important role in promoting bacterial virulence and enhancing survival during stress (64, 65). Interestingly, sphingolipids are more commonly studied in host-associated microbiomes and have been implicated in the development of metabolic disorders (66, 67). One trait associated with multiple-resilient bacterial families, including *Acetobacteraceae*, *Rhizobiaceae*, *Oxalobacteraceae*, and *Rhodospirillaceae*, was broad antibiotic/antimicrobial resistance (68–71). *Rhizobiaceae* and *Oxalobacteracea* are known to produce exopolysaccharides (EPS), which is a mechanism shown to protect bacteria from various factors, including predation and the effects of antibiotics (69, 70). *Acetobacteraceae* are also known to metabolize diverse substrates, which may confer a competitive advantage (68, 72). Another trait associated with multiple-resilient bacterial families was the ability to form growth structures such as biofilms or prosthecate cells (*Microbacteriaceae*, *Hyphomicrobiaceae*, *Oxalobacteraceae*, *Acetobacteraceae*, and *Rhodospirillaceae*) (68, 70, 71, 73, 74). Growth structures could help these bacteria keep their territory during invasions. The fungal families *Trichocomaceae*, *Herpotrichiellaceae*, and *Sporormiaceae* were both invasive and resilient, and *Phaeosphaeriaceae* were invasive (Fig. 5b). *Trichocomaceae* are known for their aggressive colonization strategies and production of mycotoxins (75). *Phaeosphaeriaceae* and *Sporormiaceae* are both known to produce antimicrobial products (76–78). Antimicrobial production is also a well-known characteristic of the resilient bacterial family *Microbacteriaceae* (73). The role of antimicrobial resistance or production as a biotic interaction mechanism contributing to success of the strongest bacterial and fungal competitors merits further study.

Our study has several limitations. While using soil suspensions as inocula instead of defined consortia has the advantage of adding relevant complexity relevant to natural systems, it creates uncertainty about the taxa in the system, in particular, those with low abundance that are sporadically detected by sequencing. This approach thus lacks control of factors such as microbial richness or diversity that may alter invasion dynamics (17, 19, 79). Because exhaustive sampling of complex resident communities is not possible, some uncertainty in labeling taxa as “invaders” is inevitable. In addition, impacts of invasions were measured at a single time point, but impacts may change over time. For example, invaders might persist below detection limits until a later opportunity in community succession allows them to flourish. Also, invaders were found primarily in low abundance; thus, their perceived success may be ephemeral. This points to the need to trace invasion outcomes over longer time frames. Longer tracing would not only provide greater insight into biotic filtering but may also reveal inflection points in the relative importance of biotic filtering and propagule pressure if these factors exert impacts over different timescales. In addition, future work is needed to delve into the specific types of biotic interactions (i.e., competition, facilitation, etc.) that are driving changes in microbial composition and ecosystem functioning following microbial invasion events.

### Conclusions.

The goal in engineering microbial communities is to alter microbiome functioning via the introduction of invaders. Our results suggest that a decision tree for probiotic design should start by considering characteristics of the target environment that may influence biotic interactions. This might also include the addition of resources (e.g., prebiotics or synbiotics) that may support microbial invasions (80). The next step is to consider biotic interactions, in particular, the ability of invaders to establish, which varies by organism type (i.e., bacteria versus fungi) and their associated traits. Delivery parameters (dose and frequency) may be considered the last measure to increase invasion success. Probiotic development is a booming industry, where market values for human and animal products alone are expected to exceed $50 billion by 2022 (81), while interest in plant probiotics is also increasing (82–84). A better understanding of fundamental principles that enhance the establishment and resilience of microbial inoculants has the potential to increase successful engineering of microbial communities for applications in human health, agriculture, bioenergy, and biotechnology.

## MATERIALS AND METHODS

### Phase I.

**(i) Microbial soil suspensions.** Soil was collected from four disparate locations, (35°01′49.4″N 106°03′03.9″W [soil 1], 38°18′36.0″N 109°16′48.0″W [soil 2], 35°58′41.430″N 79°05′39.087″W [soil 3], and 35°06′14.4″N 106°36′17.2″W [soil 4]) to obtain diverse microbial communities. For each soil, a microbial suspension was created by making a 1:100 soil dilution in a phosphate-buffered saline (PBS) and NH_4_NO_3_ (1 mg/ml) solution. Specifically, 5 g of soil was added to 45 ml of PBS and vortexed to mix. After shaking, the solution was allowed to settle for 5 min to remove large soil particulates and then 20 ml of the supernatant was transferred to 180 ml of PBS and NH_4_NO_3_ (final concentration, 1 mg/ml). This 1:100 soil dilution was created to reduce soil chemistry effects, and the microbial suspension was used as an inoculum to microcosms described further below.

**(ii) Microcosm construction.** We constructed microcosms of two different environmental types. One environment was a relatively nutrient-rich environment consisting of R2A agar medium containing diverse carbon sources, and the other environment was relatively nutrient poor consisting of milled ground pine (Pinus ponderosa) litter on sand. Microcosms consisted of 125-ml serum bottles with either 10 ml of R2A agar that was pipetted into sterile bottles while molten or 0.1 g of pine litter on 5 g of sand autoclaved three times for sterilization.

**(iii) Resident community microcosms.** Two of the initial four soil dilutions, soil 1 and soil 2, were randomly chosen to create resident community microcosms. For each soil, 1.3 ml of each microbial suspension was used to inoculate 63 replicate microcosms of each environmental type (R2A agar and pine litter), for a total of 252 microcosms. Microcosm lids were covered with aluminum foil and placed in a 25°C incubator in the dark for 30 days to allow the microbial communities from soil to establish in the novel environments (R2A agar or pine litter/sand). Previous work using similar methods has demonstrated that microbial richness in microcosms is greatly reduced compared to that in original soil samples, confirming that environmental filtering occurs over this time period (100). To maintain hydration, 0.5 ml of sterilized H_2_O was added to each microcosm weekly. At the end of 30 days, three microcosms of each type (environment-by-resident community) were destructively sampled for DNA sequencing. To do so, 5 ml of H_2_O was added to the microcosms. For R2A microcosms, a scraper was used to scrape the biofilm into solution. Microcosm material was gently vortexed for 5 s and swirled for 30 s to homogenize the mixture. A 2-ml aliquot was archived at −80°C for subsequent DNA extractions and sequencing. The remaining 240 microcosms were kept intact to use as resident microcosms in phase II. Microbial communities developed over 30 days in microcosms from soil 1 and soil 2 are here referred to as model communities, MC1 and MC2, respectively.

**(iv) Invader inocula.** Four invader inocula were derived from the four initial soil communities (soils 1 to 4) preadapted to the microcosm environments in phase I exactly as for the resident communities. For each soil at the start of phase I, 1.3 ml of the 1:100 soil microbial dilution was added to each of 15 microcosms of each environmental type (*n* = 120 microcosms total), and microcosms were incubated for 30 days as described for resident communities. At the end of phase I, this set of microcosms was used to create the four invader inocula for phase II, labeled MC1, MC2, MC3, and MC4. To create the invader inocula, samples were first suspended in 5 ml of liquid (H_2_O or R2A medium) using the same method described above for the resident community DNA sampling. For each inoculum type (community-by-environment type), the suspensions from 15 replicate microcosms were combined, yielding the 8 inocula. During the combination step, suspensions from pine litter microcosms were filtered with a 40-μm filter to remove pine litter. We did not attempt to standardize the biomass of these inocula, as previous work with pine litter microcosms has demonstrated that effects of initial differences in microbial biomass on ecosystem functioning are minimal after a 30-day incubation (85). For R2A invader inocula, the microbial abundance of each was roughly estimated using optical density (OD) measurements, and the communities were diluted to a common OD measurement.

**(v) Dose and frequency treatments.** To create different dose treatments, each inoculum type (*n* = 8) was split into three portions. One portion was used for the high-dose treatment (detailed further below). The second aliquot was diluted 1:4 and used as the low-dose treatment. This dilution ratio was chosen to minimize the impacts of dilution on composition in the complex communities and to parallel the frequency treatments (1× and 4×). The third aliquot was autoclaved for use as a killed control to account for “invasion” effects that arise solely from addition of necromass nutrients. Although autoclaving can alter the availability of specific nutrients, an autoclaved microbial suspension can nonetheless provide a baseline for the scenario where an invader inoculum is entirely consumed by the resident community microbes. Subsamples of the initial phase II invader inocula were stored along with the final phase I/initial phase II resident microbial community samples at −80°C until subsequent DNA extractions and sequencing.

### Phase II.

We used a crossed experimental design to test the effects of invasion frequency (1× or 4×) and dose (high [1×] or low [0.25×]) in determining microbial community composition and functioning (see Fig. 1). Invasions were performed by adding 0.5 ml of an invader inoculum (MC1, MC2, MC3, or MC4) into each of the resident microcosm types (MC1 and MC2) with three replicates for each treatment type (*n* = 240 total microcosms). For cases where the invader matched the resident (e.g., MC1 plus MC1), the microcosms were labeled as resident-resident controls to account for effects of perturbing resident communities with addition of more biomass. Resident microcosms received a high dose or low dose of the invader inoculum on day 1 of phase II. In addition, as a control, some resident microcosms received a killed high dose of invader inoculum. Microcosms were then sealed and placed in a 25°C incubator in the dark. CO_2_ was measured using an Agilent Technologies 490 Micro gas chromatographer (GC) on days 2, 5, 9, 16, 21, and 30. Headspace was replaced with ambient sterile-filtered air after measurements to prevent oxygen depletion and CO_2_ buildup.

**(i) 4× frequency treatment.** Approximately weekly (days 9, 16, and 23), the 4× frequency treatment microcosms received 0.5 ml of the same invader inoculum as on day 1 (stored at 6°C), delivered via sterilized syringes. To minimize manipulation differences, the 1× frequency treatments received a corresponding 0.5-ml blank aliquot of sterile H_2_O for the pine litter environment or sterile R2A medium for the R2A environment. For the killed-dose controls, introductions were performed at the 4× frequency to provide the most conservative baseline. Although invader inocula were stored at 6°C between uses for the 4× frequency treatment, we acknowledge that some growth of psychrophilic organisms may have occurred during the 23-day total storage.

**(ii) Destructive sampling.** After the final day-30 CO_2_ measurement, microcosms were destructively sampled using the same approach as in phase I. A 1.5-ml aliquot of the microbial suspension from each microcosm was archived at −80°C for DNA extraction. The remaining 3.5 ml was filter sterilized (0.2-μm filter) and stored at −20°C for dissolved organic carbon (DOC) measurements. DOC concentration was measured on an OI Analytical model 1010 wet oxidation TOC analyzer (Xylem Inc., Rye Brook, NJ, USA).

### DNA extractions and microbial community sequencing.

DNA extractions were performed with a PowerSoil DNA extraction kit (MO BIO). The standard protocol was used with the exception that 0.5 ml of homogenized liquid sample was used per extraction. Taxonomic profiling was performed by sequencing bacterial 16S rRNA and fungal 28S rRNA genes. The V3-V4 region of bacterial (and archaeal) 16S rRNA genes was amplified using primers 515f-R806 (86), and the D2 hypervariable region of fungal 28S rRNA gene was amplified using primers LR22R (87) and LR3 (88). PCR amplifications for bacteria and fungi were performed using previously described methods (87) and are described further in Text S1 in the supplemental material. Samples were sequenced on an Illumina MiSeq platform with PE250 chemistry at Los Alamos National Laboratory. Unprocessed sequences are available through NCBI’s Sequence Read Archive (PRJNA557183).

10.1128/mBio.02089-20.1 TEXT S1 Additional methods pertaining to DNA sequencing, microbial composition of residents versus invaders, and links between microbial community composition and functioning. Download Text S1, DOCX file, 0.1 MB. Copyright © 2020 Albright et al. 2020 Albright et al. This content is distributed under the terms of the Creative Commons Attribution 4.0 International license.

### Microbial community sequence analysis.

Bacterial and fungal sequences were merged with PEAR v 9.6 (89), quality filtered to remove sequences with 1% or more low-quality (q20) bases, and demultiplexed using mothur (90) allowing no mismatches to the barcode or primer sequence. Further processing was undertaken with UPARSE (91). Sequences with an error rate greater than 0.5 were removed, remaining sequences were dereplicated, singletons were excluded from clustering, OTU clustering was performed at 97%, and putative chimeras were identified *de novo* using UCHIME (91). Previous analyses have shown congruent ecological patterns with use of OTUs versus exact sequence variants (ESVs) for delineating microbial taxa (92). Furthermore, OTU clustering at 97% provides a more conservative estimate of overlaps between introduced and resident taxa. Bacterial and fungal OTUs were classified using the Ribosomal Database Project (RDP) classifier (93). Using the OTU matrices for final communities (*n* = 240), we rarefied bacterial and fungal profiles to the lowest common number of sequences (*n* = 1,020 and *n* = 1,262, respectively) and calculated Bray-Curtis distance matrices (94) and diversity metrics (richness and Shannon diversity) (95).

In addition to analyses at the community level, we examined how invasions altered microbial composition at the taxa (OTU) level (Text S1). The four model microbial communities contained both common and unique taxa; therefore, we looked at the distribution of taxa across the different initial communities and how that distribution changed with the addition of each invader community. We split analyses by environmental type. For each invader-by-resident community combination, we compared the presence/absence of OTUs in the final invader-resident samples (invader-resident) to OTUs in the introduced inoculum samples (invader initial) and the resident control samples (resident control). The resident control included resident-resident final samples, resident-killed final samples, and initial resident samples. Overall, this led to analysis of 12 unique introduction events, including 6 per environment type (MC1 into MC2, MC3 into MC2, MC4 into MC2, MC2 into MC1, MC3 into MC1, and MC4 into MC1). For each event, we calculated the percentage of the total OTUs found in each category, including “resident control only” (nonresilient), “invader inoculum only” (noninvasive), “invader-resident final only” (undetermined origin), “resident control plus invader-resident final” (resilient), “invader inoculum plus invader-resident final” (invasive), “invader inoculum plus resident control” (nonresilient), and “all” (common). In addition, for each event, we also calculated the relative abundance of OTUs at the end of phase II across the common, resilient, invader, and undetermined origin categories. To account for the potential influence of rarefaction, we used average values generated from 100 rarefactions of the initial OTU tables, and we compared sequencing depth by using rarefaction cutoffs of 1,020/1,262 (bacteria/fungi), 5,000, and 10,000 sequences (Table S2). Sequence depth did not impact the results (G-test of independence), and results of rarefaction to 1020/1262 sequences are presented in the text and in Fig. 4. OTU tables and R codes for this analysis are available at (https://github.com/mbnalbright/Community-Invasion).

### Competitive strength of taxa.

All of the invaders and resilient taxa OTUs from each of the 12 introduction events were aggregated to assess competitive strength. Across the 12 invasion events, there were numerous instances where in one event (e.g., MC1 invading MC2), a taxon was resilient, and in a separate event (e.g., MC2 invading MC1), the taxon was invasive. Both outcomes represent successful competition. For each taxonomic family, a strong competitor score was calculated as

(∑i=1kNiFinalNiInitial)×k,

where *k* is the total number of invasive and resilient OTUs in a family, and *N* is the average abundance of an OTU in the final and initial communities. OTUs with <70% taxonomic confidence identity at the family level were excluded. For a conservative assessment of the strong bacterial competitors, we focused on families with competitor scores of greater than 50 found in both the agar and litter environments. As a final step, for each family, we averaged competitor scores across the two environment types. We used the same process for fungi, but fungi data were only available for the litter environment. We evaluated weak competitors in a similar fashion but used noninvasive and nonresilient OTUs and simplified the competitor score to the sum of *N_iInitial_* × *k*. Results for bacteria were filtered by families with weak competitor scores greater than 50 found in both agar and litter environments, and fungi were filtered by weak competitor scores greater than 50. Again, to account for the potential influence of rarefaction, we used average competitor scores generated from 100 rarefactions of the initial OTU tables. In addition, we tested the influence of changing rarefaction cutoffs at the cutoff values of 1,020/1,262 (bacteria/fungi), 5,000, and 10,000. The vast majority (>97%) of identified strong and weak taxonomic families were unaffected by the rarefaction cutoffs. Results are presented for the 1,020/1,262 cutoff, but we excluded the small number of families that were not found at all three rarefaction cutoffs and/or across multiple rarefactions in a single cutoff.

### Statistical analysis.

First, using the end communities for th*e* phase II experimental treatments, we tested the impact of invaders on community composition and ecosystem functioning by comparing the influence of invader (e.g., MC2-MC1) compared to that of control treatments (e.g., killed MC1-MC1 and MC1-MC1) on community composition and ecosystem functioning across two environments (agar and litter). Here, we used a one-way ANOVA for univariate metrics (i.e., CO_2_ production, DOC production, richness, and Shannon diversity) and pairwise permutational multivariate ANOVAs for multivariate metrics (i.e., bacterial and fungal composition).

Next, using only final invaded end communities (*n* = 144) excluding controls (see Fig. 1), we looked at what factors most impacted microbial composition and ecosystem functioning following invasion events. Here, our phase II experimental treatments included four factors: invader community (MC1, MC2, MC3, and MC4), resident community (MC1 and MC2), dose (high [1×], low [0.25×]), and frequency (1× addition, 4× addition), which we examined across two environment types (agar, litter). To test the impact of treatment factors and estimate the variance explained by each treatment in driving variation in univariate metrics (i.e., CO_2_ production, DOC production, richness, and Shannon diversity), we used a multifactorial ANOVA design with all four manipulated variables as main fixed factors. We tested the effects of the main factors as well as interaction terms (dose × frequency × invader community × resident community). Analyses were performed on full models and then reduced models were run with only the significant factors. Analyses were performed separately for each environmental type. The ANOVA analyses were conducted in the R software environment (96). To assess the contribution of treatments in driving variation in bacterial and fungal community composition, we performed a permutational multivariate analysis of variance (PERMANOVA) (97), using the same factors as with the univariate tests. Using results from the reduced models, we estimated the percent variation that could be attributed to each significant term for both the ANOVA (98) and PERMANOVA (as described in reference 99).

### Data availability.

All raw 16S rRNA and LSU rRNA unprocessed sequence data are available through NCBI’s Sequence Read Archive (PRJNA557183). OTU tables and R scripts for the invader analysis are available at (https://github.com/mbnalbright/Community-Invasion).
