# Supplementary material for: Biotic Interactions Are More Important than Propagule Pressure in Microbial Community Invasions
Source: mBio. 2020 Oct 27;11(5):e02089-20. doi: 10.1128/mBio.02089-20 (PMC7593967; doi:10.1128/mBio.02089-20)
Supplement: TABLE S1 [file mBio.02089-20-st001.docx]

**Table S1.** Factors driving variation in ecosystem functioning (CO_2_ and DOC accumulation) and bacterial and fungal richness and composition in litter and agar environments
