# Supplementary figures and images for: Biotic Interactions Are More Important than Propagule Pressure in Microbial Community Invasions
Source: mBio. 2020 Oct 27;11(5):e02089-20. doi: 10.1128/mBio.02089-20 (PMC7593967; doi:10.1128/mBio.02089-20)

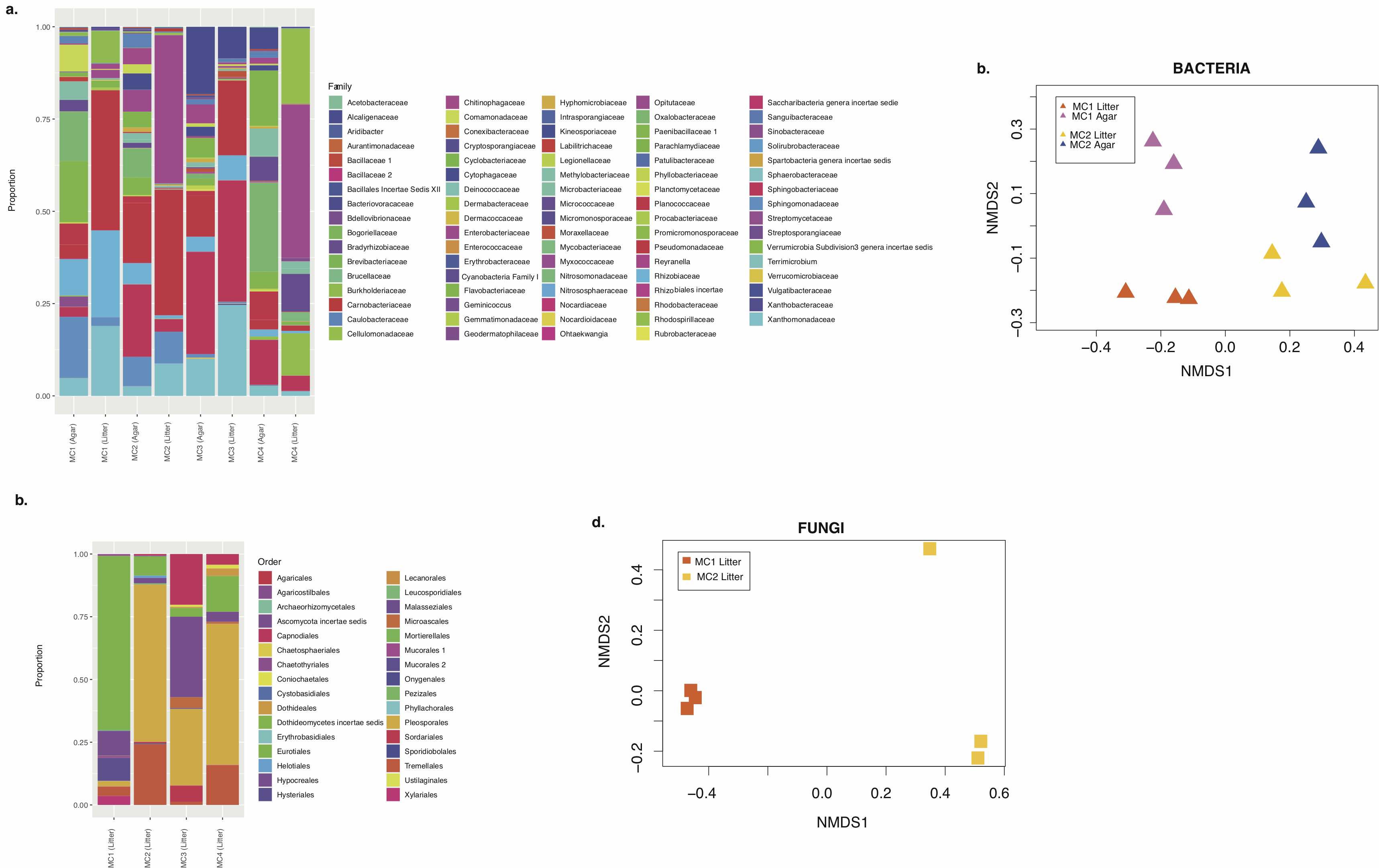

Supplement: FIG S5 [file mBio.02089-20-sf005.jpg]
