# Supplementary material for: Biotic Interactions Are More Important than Propagule Pressure in Microbial Community Invasions
Source: mBio. 2020 Oct 27;11(5):e02089-20. doi: 10.1128/mBio.02089-20 (PMC7593967; doi:10.1128/mBio.02089-20)
Supplement: TEXT S1 [file mBio.02089-20-s0001.docx]

**Biotic interactions are more important than propagule pressure in microbial community invasions**

Michaeline B.N. Albright^1^, Sanna Sevanto^2^, La Verne Gallegos-Graves^1^, John Dunbar^1^

^1^Bioscience Division, Los Alamos National Laboratory, Los Alamos, New Mexico

^2^Earth and Environmental Sciences Division, Los Alamos National Laboratory, Los Alamos, New Mexico

Michaeline B.N. Albright^1^

Email: [malbright@lanl.gov](mailto:malbright@lanl.gov)

**Supplementary Materials Text**

*DNA sequencing*

PCR amplifications for bacteria and fungi were performed using a two-step approach (84). In the first PCR, sample barcoding was performed with forward and reverse primers each containing a 6-bp barcode; 22 cycles with an annealing temperature of 60^o^C were performed (86). The second PCR added Illumina adaptors over 10 cycles with an annealing temperature of 65^o^C. Amplicon clean-up was performed with a Mobio UltraClean PCR clean-up kit, following manufacturer’s instructions with the following modifications: binding buffer amount was reduced from 5X to 3X sample volume, and final elutions were performed with 50 µl Elution Buffer. Following clean-up, samples were quantified with an Invitrogen Quant-iT^TM^ ds DNA Assay Kit on a BioTek Synergy HI Hybrid Reader. and pooled at a concentration of 10 ng per sample. A final clean-up step was performed on pooled samples using the Mobio UltraClean PCR clean-up kit.

*Microbial composition of residents versus invaders*

The four Phase II inoculum communities were significantly different from each other (pairwise permutation MANOVAs; Figure S6). Replicate resident communities within an environment from the initial *Phase II* sampling were more similar to each other than to other resident communities in that environment (Figure S6). The four complex model microbial communities contained both common and unique taxa, therefore we looked at the distribution of taxa across the different initial communities, and how that distribution changed with the addition of each invader community. Here, we split analyses by environmental type, as our community analyses showed that the environment played a large role in structuring final microbial composition.

*Assessing links between microbial community composition and functioning*

Using community composition data from all samples including controls (n=240), we tested the correspondence between microbial (fungal and bacterial) community and functional metrics (CO_2_ and DOC) using Pearson’s correlations for univariate metrics (richness, diversity) (Rcorr package). In addition, in order to link microbial community composition and functioning, we grouped samples into high (1/3), mid (1/3), and low (1/3) categories based on DOC and CO_2_ values. We ran a two-way PERMANOVA using DOC and CO_2_ categories as factors, including a DOC-by-CO_2_ interactions term.

Across all samples final bacterial community composition was linked to both CO_2_ and DOC (PERMANOVA; Agar CO_2_: F_2,4_=1.9, p=0.004; DOC: F_2,4_=8.8, p=0.001; CO_2_-by-DOC: F_4,92_=1.3, p=0.028, Litter CO_2_: F_2,4_=3.3, p=0.001; DOC: F_2,4_=6.4, p=0.001; CO_2_-by-DOC: F_4,101_=1.4, p=0.062) (Figure S7e). Bacterial richness was positively correlated with cumulative CO_2_ production in both environments, although the correlation was stronger for agar than litter (R^2^=0.72, p<0.001 and R^2^=0.29, p=0.002, Figure S7a). Bacterial richness was negatively correlated with DOC in litter (R^2^=-0.63, p<0.001) and agar (R^2^=-0.4, p<0.001) environments (Figure S7c). Fungal community composition on litter was linked to CO_2_ production, but not DOC (PERMANOVA; CO_2_: F_2,4_=3.1, p=0.002; DOC: F_2,4_=1.7, p=0.1; CO_2_-by-DOC: F_4,71_=0.9, p=0.636) (Figure S7f). The differences in CO_2_ production across the fungal communities largely corresponded to the resident community composition. Fungal richness was negatively correlated with CO_2_ production (R^2^= -0.28, p=0.007, Figure S7b) but was not significantly correlated with DOC (Figure S7d).
